# Oral Focal Mucinosis of the Lingual Gingiva in an Adolescent: A Case Report of an Uncommon Presentation With a Review of the Literature

**DOI:** 10.7759/cureus.111745

**Published:** 2026-06-29

**Authors:** Chanda Yadav, Farrukh Faraz, Arundeep K Lamba, Shruti Tandon, Deepmala Maurya, Priya Kumar

**Affiliations:** 1 Periodontics, Maulana Azad Institute of Dental Sciences, New Delhi, IND; 2 Oral and Maxillofacial Pathology, Maulana Azad Institute of Dental Sciences, New Delhi, IND

**Keywords:** adolescents, clinical diagnosis, gingival overgrowth, oral focal mucinosis, rare case report

## Abstract

Oral focal mucinosis (OFM) is a benign lesion of the oral cavity. It is usually a slow-growing, painless nodular swelling commonly located on the gingiva. It has non-specific clinical features and is often confused with reactive lesions of the gingiva. It is rarely reported among adolescents. An 11-year-old girl reported with a firm, painless nodular swelling on the lingual gingiva of the lower jaw for six months. Growth was excised surgically, and histopathological examination was performed using H&E stain. Microscopic analysis revealed the presence of a myxoid stroma with spindle-shaped stellate fibroblasts and plenty of mucin, which confirmed the diagnosis of OFM. Healing was uneventful, and 12 months of follow-up did not report any recurrence. This case highlights the need to include OFM as a differential diagnosis of gingival swellings presented in adolescents. Clinicians should take OFM into account when making a differential diagnosis of gingival swellings to facilitate accurate diagnosis and appropriate management.

## Introduction

Oral focal mucinosis (OFM) is a rare, benign connective tissue entity of the oral cavity [1]. It was first characterized by Tomich in 1974 [1]. Although the exact etiology remains unclear, trauma-induced fibroblastic dysfunction leading to abnormal mucin accumulation has been proposed [2]. Clinically, it mimics reactive lesions such as fibrous hyperplasia or peripheral ossifying fibroma [3]. Histopathological examination is required for definitive diagnosis. Most cases illustrate a hypocellular myxoid stroma with a low concentration of stellate and spindle-shaped fibroblasts and low vascularity [4]. Conservative surgical excision remains the treatment of choice [5]. Recurrence after proper surgical excision is very uncommon. Most documented cases of OFM are seen in middle-aged women from the fourth decade of life [5]. It is uncommon in adolescents. Hence, we report a rare case of OFM involving the lingual gingiva in an adolescent, highlighting the diagnostic challenges associated with this uncommon presentation, its characteristic clinicopathological features, and a brief review of the literature.

## Case presentation

**Figure 1:**
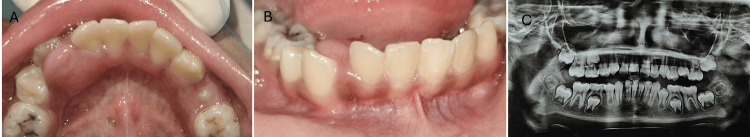
Preoperative clinical and radiographic views. (A) Lingual view showing well-circumscribed gingival enlargement. (B) Buccal view illustrating the extent of the lesion. (C) Panoramic radiograph (orthopantomograph (OPG)) demonstrating no evidence of underlying bone loss.

An 11-year-old female patient presented to the Department of Periodontics with a chief complaint of difficulty while chewing due to swelling involving the mandibular right anterior region (Figures 1A, 1B). According to the patient and her parents, the growth was first noticed as a small swelling approximately six months ago and progressively enlarged in size over time, which resulted in difficulty during mastication. On intraoral examination, a solitary and clearly defined growth involving the lingual gingiva was seen in relation to teeth 42 and 43 on the right side of the mandible. The lesion was approximately 15 x 10 mm in size. It appeared as a sessile nodular mass, pinkish in color. Palpation showed a non-tender growth with hard, bony consistency. The gingival tissue surrounding the lesion was clinically normal, and there were no signs of inflammation. An orthopantomograph (OPG) revealed no signs of bony involvement (Figure 1C).

**Figure 2:**
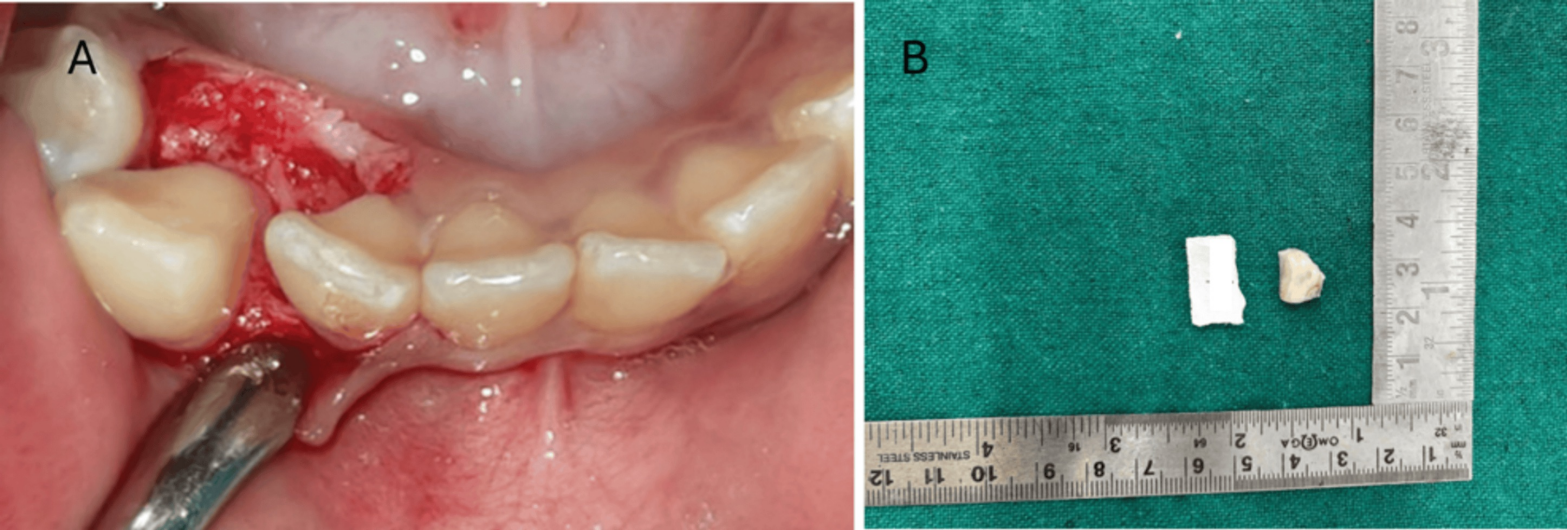
(A) Surgical excision of growth. (B) Gross specimen obtained after surgical excision.

Based on clinical presentation, a provisional diagnosis of fibroma was made. Differential diagnoses were peripheral ossifying fibroma and focal fibrous hyperplasia. Growth was completely excised following local anesthetic infiltration (Figures 2A, 2B).

**Figure 3:**
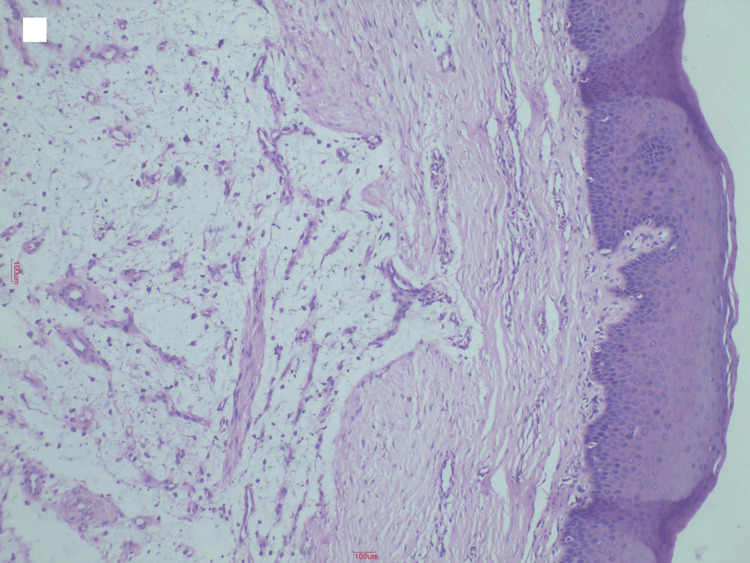
H&E-stained view (×10 magnification). The photomicrograph shows well-circumscribed myxomatous tissue covered by hyperparakeratinized stratified squamous epithelium with short and broad rete ridges.

**Figure 4:**
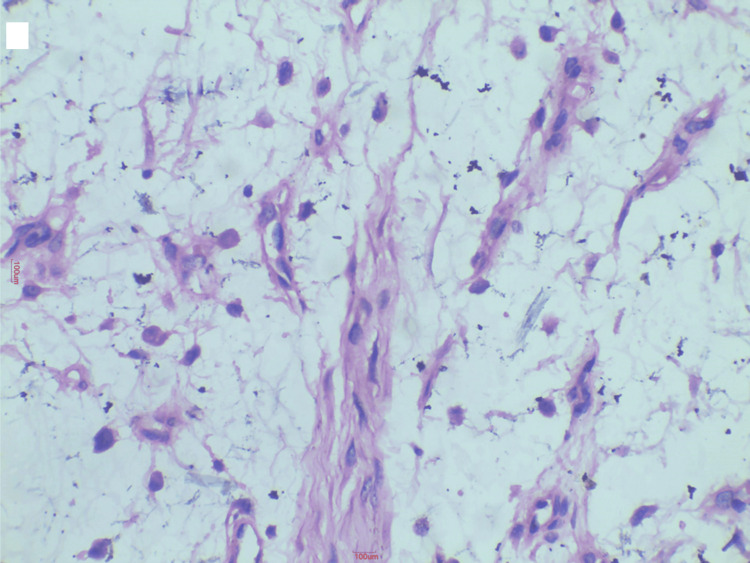
H&E-stained view (×40 magnification). The photomicrograph shows a myxomatous area with loosely arranged thin collagen fibers, plump fibroblasts, and budding capillaries.

**Figure 5:**
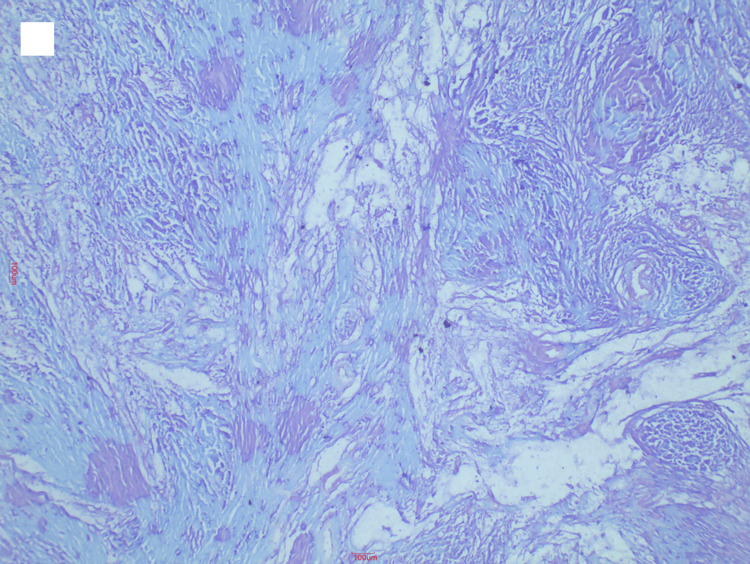
Histochemical staining with Alcian blue is strong and diffuse in OFM, which confirms the presence of abundant mucin dispersed in connective tissue. OFM: oral focal mucinosis

Microscopic evaluation of H&E-stained slides showed well-circumscribed areas of abundant myxoid connective tissue composed of loosely arranged thin fibrillar collagen fibers, plump ovoid spindle-shaped fibroblasts, and budding capillaries. The surrounding connective tissue exhibited moderately dense collagen bundles arranged in parallel with associated spindle fibroblasts and vascular channels. Minimal lymphocytic inflammatory infiltrate was observed. The overlying epithelium consisted of hyperparakeratinized stratified squamous epithelium with short and broad rete ridges. Alcian blue staining at pH 2.5 demonstrated intense blue staining, indicating abundant acidic mucin rich in hyaluronic acid. Based on these histopathological findings, a definitive diagnosis of OFM was established (Figures 3-5).

**Figure 6:**
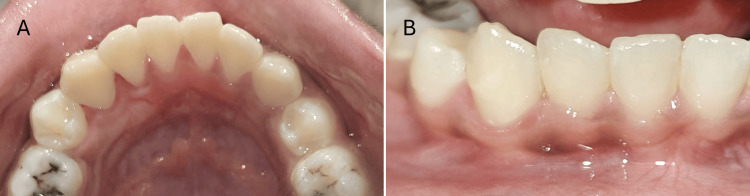
Postoperative view. (A) Lingual view at 12 months. (B) Buccal view at 12 months.

Postoperative healing was uneventful. The patient was followed for 12 months, and no evidence of recurrence was seen (Figures 6A, 6B).

## Discussion

Cutaneous focal mucinosis was first reported in 1966 by Johnson and Helwig as a benign and asymptomatic lesion that commonly appears on the face or trunk as a dome-shaped nodule [6]. OFM is an intraoral analogue of cutaneous focal mucinosis. Approximately 12 cases of OFM in adolescents have been documented in the existing literature worldwide [7-14]. Limited cases have been reported from the Indian subcontinent, emphasizing the rarity of the present case.

Thus, the current case adds valuable information to the scanty literature on OFM in younger people. Previously reported pediatric and adolescent cases are summarized in Table 1.

**Table 1:** Literature review of reported cases in pediatrics and adolescents.

Author	Country	Age	Gender	Site	Duration	Differential diagnosis	Management
Tomich (1974) [1]	USA	16	Male	Gingiva	Not available	Irritation fibroma	Excision
Aldred et al. (2003) [2]	Australia/UK	16	Female	Gingiva	4 months	Fibrous hyperplasia	Excision
Buchner et al. (1990) [7]	Israel	16	Female	Gingiva	Not available	Not available	Not available
Gnepp et al. (1989) [8]	Not available	4	Female	Hard palate	Not available	Not available	Not available
Lee et al. (2012) [9]	Australia	17	Female	Gingiva	Not available	Not available	Not available
Woo and Cheung (2015) [10]	China	2	Female	Palate	3 months	Pleomorphic adenoma, palatal exostosis	Excision
Yanaguizawa et al. (2018) [11]	Brazil/Japan	14	Female	Buccal mucosa	Not available	Fibroma	Excision
Cho et al. (2019) [12]	USA	13	Female	Gingiva	4 months	Fibroma, peripheral ossifying fibroma	Excision
Cameron et al. (2020) [13]	UK	14	Female	Gingiva	Not reported	Fibroma	Excision
Silva Cunha et al. (2021) [3]	Brazil	8	Male	Gingiva	36 months	Pyogenic granuloma	Excision
Tonkaboni et al. (2022) [14]	Iran	17	Female	Palate	1 year	Fibroma	Excision
This case	India	11	Female	Gingiva	6 months	Peripheral ossifying fibroma	Excision

The pathophysiology of OFM results from the overproduction of hyaluronic acid by oral mucosal fibroblasts; an excessive amount of hyaluronic acid causes myxoid degeneration of connective tissue. Nilesh et al. proposed traumatic stimuli as a predisposing factor for OFM [15]. It often resembles reactive lesions such as irritation fibroma or pyogenic granuloma, making preoperative diagnosis difficult. Similar to previously documented cases, the lesion in the present patient was clinically similar to a fibroma. This case highlights the diagnostic dilemma of this entity.

Histopathological features in OFM are a well-circumscribed non-encapsulated stroma of myxoid connective tissue with sparse fibroblasts that are spindle-shaped and have a high content of ground substance containing hyaluronic acid. Absence of cellular atypia as well as infiltrative growth helps to differentiate it from the odontogenic myxoma and other myxoid neoplasms. Alcian blue staining at pH 2.5 demonstrated intense blue staining of the stromal mucin, confirming the presence of abundant acidic mucopolysaccharides, predominantly hyaluronic acid. Based on the histopathological and histochemical findings, a definitive diagnosis of OFM was established.

Because of its generic clinical presentation, most of the cases that have been reported, including the present case, could not be diagnosed until histopathological examination was done. So, definitive diagnosis is substantially based on histopathological evaluation [16]. Management of OFM is primarily done by surgical excision. The prognosis is good in cases of complete surgical excision, and recurrence is rare. However, there is one reported case of recurrence on the upper lip [3]. In line with other reports, our patient showed a smooth recovery and no recurrence at 12 months of follow-up.

## Conclusions

OFM represents as a clinically elusive gingival lesion. Its asymptomatic and slow-growing nature, along with normal-appearing overlying mucosa, makes clinical recognition challenging, especially in adolescents. Histopathological examination is essential for proper diagnosis. Clinicians should take OFM into account when making a differential diagnosis of gingival swellings to facilitate accurate diagnosis and appropriate management. Although this report is limited to a single case, it highlights the importance of recognizing this rare entity and contributes to the existing literature by documenting its uncommon presentation on the lingual gingiva in an adolescent.
